# ‘In general, how do you feel today?’ – self-rated health in the context of aging in India

**DOI:** 10.3402/gha.v7.23421

**Published:** 2014-04-22

**Authors:** Siddhivinayak Hirve

**Affiliations:** Vadu Rural Health Program, KEM Hospital, Pune, India

**Keywords:** self-rated health, aging, mortality, disability, reporting heterogeneity, India

## Abstract

This thesis is centered on self-rated health (SRH) as an outcome measure, as a predictor, and as a marker. The thesis uses primary data from the WHO Study on global AGEing and adult health (SAGE) implemented in India in 2007. The structural equation modeling approach is employed to understand the pathways through which the social environment, disability, disease, and sociodemographic characteristics influence SRH among older adults aged 50 years and above. Cox proportional hazard model is used to explore the role of SRH as a predictor for mortality and the role of disability in modifying this effect. The hierarchical ordered probit modeling approach, which combines information from anchoring vignettes with SRH, was used to address the long overlooked methodological concern of interpersonal incomparability. Finally, multilevel model-based small area estimation techniques were used to demonstrate the use of large national surveys and census information to derive precise SRH prevalence estimates at the district and sub-district level. The thesis advocates the use of such a simple measure to identify vulnerable communities for targeted health interventions, to plan and prioritize resource allocation, and to evaluate health interventions in resource-scarce settings. The thesis provides the basis and impetus to generate and integrate similar and harmonized adult health and aging data platforms within demographic surveillance systems in different regions of India and elsewhere.

This thesis takes place in the context of a rapidly aging population in India as seen from a persistently high (>30%) percentage decadal growth in its elderly population over the last few decades despite a steady decline in the overall percentage decadal growth from 24.8% in 1971 to 17.6% in 2011. India's elderly population has grown four-fold in the last 50 years and with current trends projected to triple to about 300 million by 2050 (1). India is set to alter its status from that of a young population to an aging population by 2030, yet it may be debated whether it has the requisite policies and infrastructures in place to address the growing needs and challenges of the elderly (2). The majority of elders are outside the social safety net, and they face economic, health, and emotional insecurity and inequity that pose a challenge to an already overburdened societal system (3). The new millennium has seen a concerted global effort to mainstream aging into the development agenda, and countries have agreed to link questions of aging to frameworks for social and economic development and human rights (4). Yet, a decade later, aging population concerns suffer lack of attention, resources, and political visibility. Questions that need answers to formulate policies on successful aging are seemingly endless and complex. Additionally, population aging research has largely been in the domain of demographers and economists focused on living arrangements of the elderly (5), risk factors, disease, and disability arising from obesity and age-related degenerative conditions (6, 7). Until recently, the lack of a globally harmonized data infrastructure that could simultaneously explore all the key life domains of the elderly – work history, leisure, income, wealth, social and emotional securities, health behaviors, disease, disability, health care utilization, cognition, ability to perform activities of daily living, life satisfaction, quality of life (QOL), and subjective wellbeing – has been a major drawback to a more holistic approach to research on successful population aging.

Self-rated health (SRH) is a complex latent construct commonly used to assess health and wellbeing (8). It refers to a survey technique where individuals assess their own health by answering a single global health question ‘In general, how would you rate your health today?’ or a series of questions such as ‘In the last 30 days, how much difficulty did you have in moving around?’, ‘… with remembering things?’, and so on that are typically structured on a Likert scale (9). Though the exact wordings and response options of SRH have varied between surveys making direct comparisons difficult, it essentially assesses the same phenomenon across different settings (10). The individual chooses a self-rating response by a cognitive process that is inherently subjective as well as contextual – the physiological, biological, and emotive experiences and expectations influenced by the contextual social environment (11). It is an all-inclusive, sensitive yet non-specific measure that assesses health and predicts health outcomes in ways that are still unclear (12). Such a simple, yet poorly understood, cost-effective self-perception measure has immense practical utility in assessing elderly health and health care (13, 14) substituting other more expensive and invasive measures in resource-scarce settings.

## Scope and setting

**Fig. 1:**
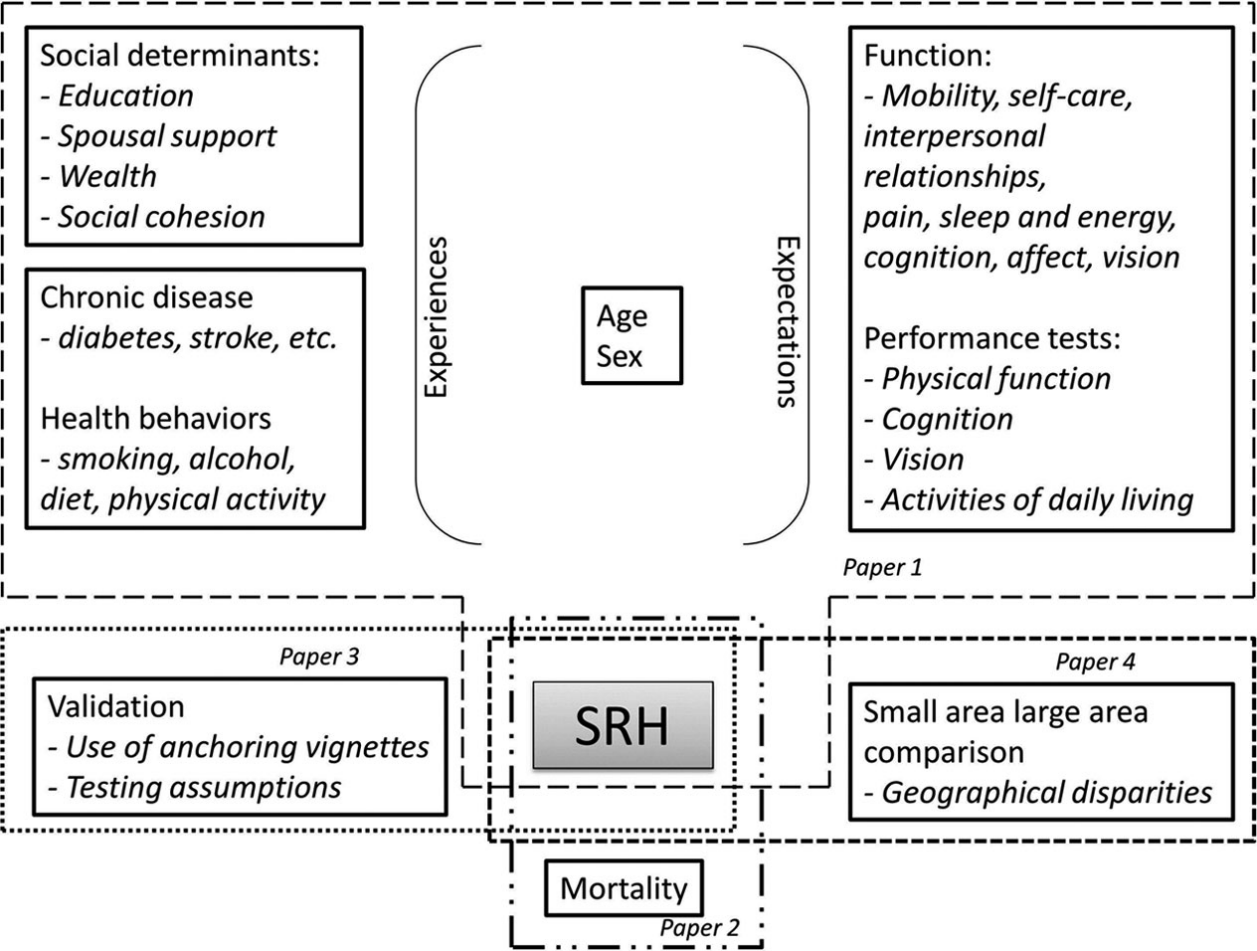
Framework for scope of the thesis.

Figure 1 defines the scope of this thesis. It uses newer advances in statistical approaches to explore three distinct yet interlinked thematic tracks centered on SRH as an ‘outcome’ measure; SRH as a ‘predictor’ variable; and SRH as a ‘marker’ variable. Table 1 summarizes the thematic tracks. The first theme uses the structural equation modeling approach (15) to understand pathways through which the social environment, functional disability, and disease experience influence SRH as well as mediate the effects of age and sex on SRH. The second theme builds further on examining the role of SRH and the influence of disability in predicting mortality using the conventional Cox proportional hazards modeling approach (16). The third theme addresses the methodological concern of interpersonal comparability of SRH. It uses a hierarchical ordered probit (HOPIT) modeling approach to combine information from anchoring vignettes to identify and correct self-rating responses for reporting heterogeneity (17). Finally, a fourth theme validates the use of national surveys to derive precise estimates of SRH at the district and sub-district level using random effects model based small area estimation techniques (18).

**Table 1:** Overview of the thematic tracks for the thesis

	Paper 1	Paper 2	Paper 3	Paper 4
Title	Unpacking self-fated health and quality of life (QOL) in older adults and elderly in India: A Structural Equation Modeling approach	Does self-rated health predict death in adults aged 50 years and above in India? Evidence from a rural population under health and demographic surveillance	Evaluating reporting heterogeneity in self-rating health responses among adults aged 50 years and above in India – an anchoring vignettes analytic approach	Self-rated health: small area–large area comparisons among older adults at the state, district, and sub-district level in India
Objective	To understand pathways that influence SRH	To examine the predictive role of SRH and mortality	To improve inter-personal comparability of self-reported measures of health	To compare directly and indirectly derived small area estimates
Data sets	Full SAGE (Vadu) + HDSS (Vadu)	Short SAGE (Vadu) + HDSS (Vadu)	Short SAGE (Vadu) + Full SAGE (Vadu) + HDSS (Vadu)	Short SAGE (Vadu) + Full SAGE (India) + HDSS (Vadu) + Census 2011 (India)
Statistica methods	Structural Equation Model	Cox Proportional Hazard Model	Hierarchical Ordered Probit Model	Multilevel Logistic Regression Model, Bayesian Logistic Regression Model
Statistical software	Linear Structural Relations (LISREL) 8.8	Stata 11	Stata 11 R	Stata 11 Windows Bayesian under Gibbs Sampling (Win BUGS) 14
Main findings	Higher educated, richer had significantly higher levels of social cohesion that in turn had significantly better QOL and SRH; Direct effect of socioeconomic status on QOL/SRH was not significant. Older age had significantly lower QOL and SRH mediated through functional ability	Men with poor SRH had a significant three-fold increase in mortality hazard; not significant for women; Lack of spousal support and disability significantly increased mortality hazard	Strong evidence of reporting heterogeneity largely driven by age, sex and socioeconomic status; Higher socio-economic status more demanding, older ages less demanding in their self-assessment of health; Individuals understood vignettes in the same way; Individuals used different thresholds while rating self and rating vignettes	Indirect synthetic estimate had poor approximation while Best Linear Unbiased Prediction (BLUP) and Hierarchical Bayes (HB) estimate had good approximation to direct survey estimate

**Fig. 2:**
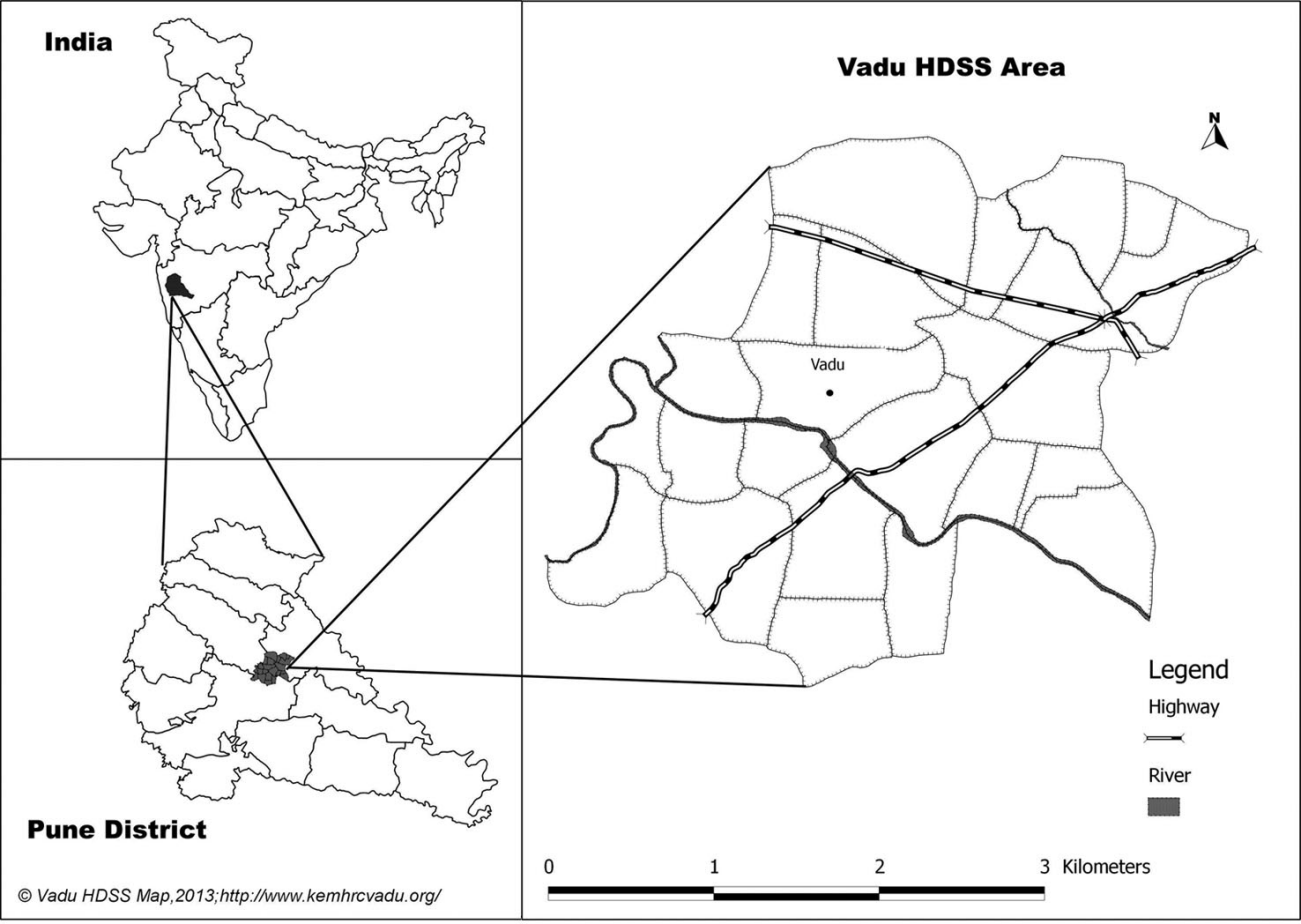
Vadu health and demographic surveillance area in rural Pune district, India. Source: Vadu HDSS, KEM Hospital Research Center, Pune.

The thesis is grounded in the Vadu community comprising of more than 100,000 population residing in 22 villages in the rural Pune district in Maharashtra in India (Fig. 2). The Vadu community has been under health and demographic surveillance (HDSS) since 2002 wherein a biannual household census enumerates all births, deaths, and migrations and ascertains cause of death. The World Health Organization (WHO) study on global aging and adult health (SAGE) was administered to a multistage stratified cluster random sample of 7,150 individuals aged 50 years and above at the national level in 2007–08. It was also administered to a simple random sample of 321 individuals aged 50 years and above from the Vadu community. SAGE collected information on household and individual sociodemographic characteristics, work history, SRH, functional health state, health behaviors, chronic illnesses, health care utilization, social cohesion, QOL, and subjective wellbeing (Appendix) (19). Furthermore, an abridged version (SRH, functional health state, QOL, and subjective wellbeing) of the SAGE survey was administered to an independent random sample of 5,432 individuals aged 50 years and above in the same Vadu community (20). Individuals graded their ability to perform tasks in eight functional domains of health (mobility, affect, self-care, cognition, pain, interpersonal relationships, sleep and vision). Each domain included two self-rating questions – one for a lower and another for a higher level of functional ability. A total of 10 anchoring vignettes in two functional domains were administered to each individual at random. After each vignette, the same question as the two self-rating questions was asked. Individuals rated their self and vignette assessments on a five-point ordinal scale of increasing difficulty. Three additional measures – WHO Health State score, WHO Disability Assessment Schedule (DAS) score, and the WHO QOL score – were derived from the questions on limitations in functional ability and subjective wellbeing. The SAGE dataset was further enhanced by linking it with the HDSS dataset for individual and household sociodemographic characteristics and to identify deaths among study participants in the 4 years subsequent to the SAGE survey.

The sociodemographic characteristics of the individuals included in the abridged version and the full version of the SAGE survey were similar to that of the Vadu population from which they were sampled. However, the Vadu SAGE participants were significantly older and less educated compared to the participants of the national SAGE survey. Table 1 summarizes the main findings of the thesis.

## SRH as an outcome

Globally, there is a large body of literature on the psychosocial and socioeconomic determinants of SRH. It is known to worsen as age advances, and women are more likely to report poor SRH compared to men (21–23). Limitations in physical and mental function – sleep, mobility, cognition – are strongly associated with poor SRH (24–26). Widowhood, lack of education, and lower levels of social networking and social cohesion are known to be associated with poor SRH (27–31). Health behaviors and absence of chronic illness predict physical function and contribute to good SRH in old age (32–34). However, the pathways through which these variables influence SRH are not known.

**Fig. 3:**
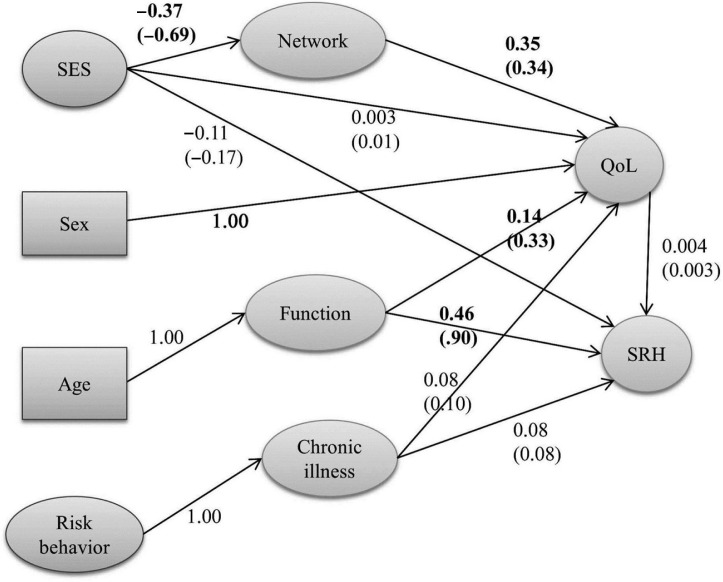
Structural equation model for SRH. Standardized coefficients (effects) are in parenthesis. Latent variables are depicted as ovals and observed variables as rectangles. Final model *χ*^2^=409.87, *df*=271; RMSEA = 0.041. Source: Hirve et al., (35).

We tested a simple theory where individual sociodemographic characteristics influenced QOL and SRH through intermediate mediators such as functional ability and social cohesion (Fig. 3) (35). Older individuals reported significantly poorer SRH and QOL that was mediated through limitations in functional ability. Individuals with higher education and with a regular income had higher levels of social networking/social cohesion that in turn had a positive effect on QOL and SRH. However, the direct effect of socioeconomic status on SRH or QOL was not significant. Smoking or tobacco consumption was associated with at least one chronic illness which in turn was associated with poor QOL and SRH – this association was, however not significant.

## SRH as a predictor

A strong association between poor SRH and risk of mortality, independent of age, sex, income, education, social networking, health behavior, and chronic disease, is consistently reported from Europe and North America (36–38) and in Asia (39, 40). A meta-analysis of 22 studies shows a two-fold increase in all-cause mortality for persons who report poor SRH (41). There is some evidence from developed countries that high levels of disability and morbidity, and poor physical and cognitive function may influence this association (42, 43). It is unclear to what extent disability and the social environment alters the predictive ability of SRH on mortality.

**Fig. 4:**
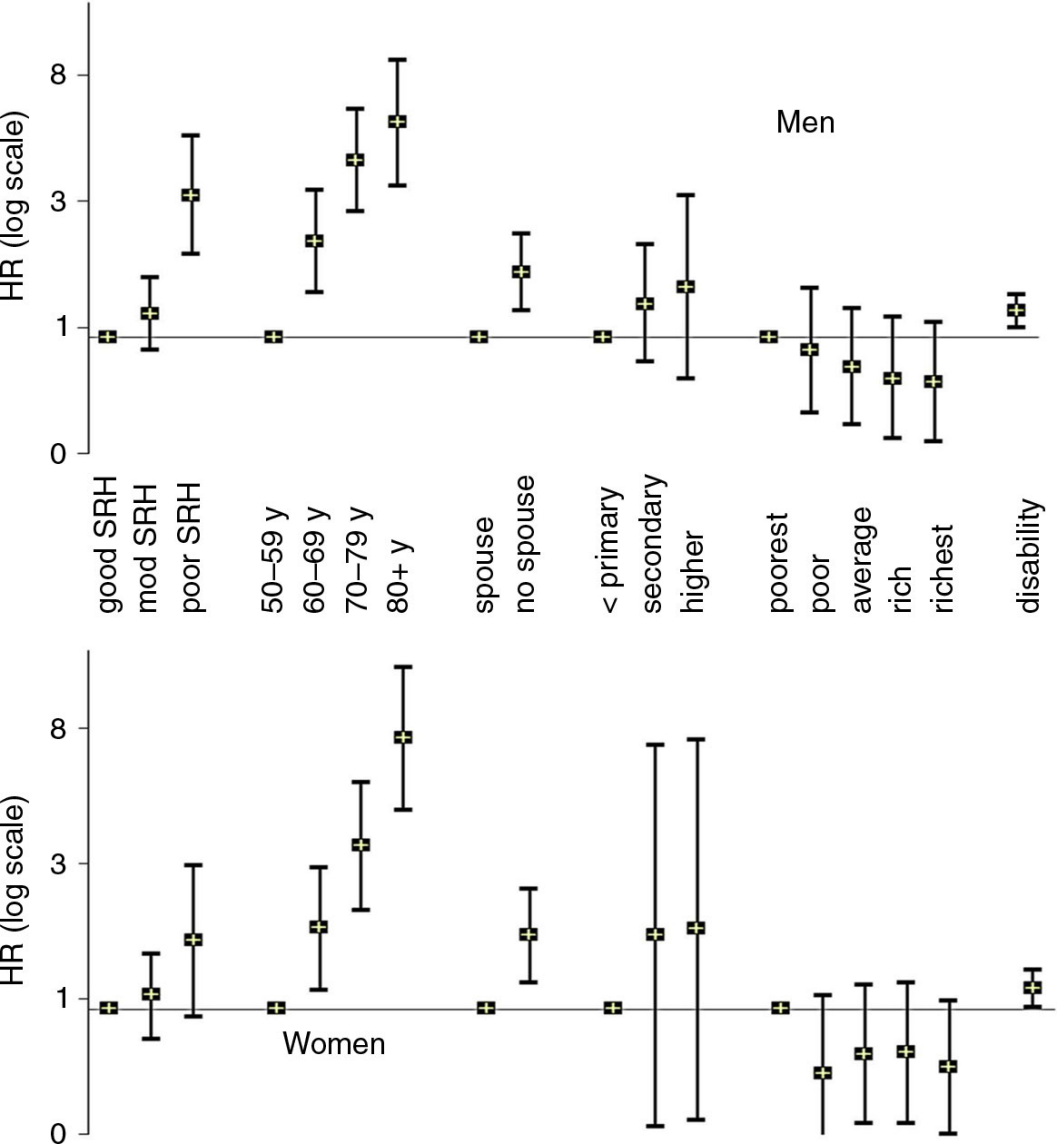
Hazard ratio for mortality. Reference categories are ‘good/very good SRH’, ‘50–59 years age’, ‘spousal support’, ‘primary or less education’, and ‘poorest socioeconomic quintile’.

In our study, men who reported poor/very poor SRH had a three-fold higher hazard for mortality compared to those who reported good/very good SRH independent of age, disability, and socioeconomic characteristics (44). A similar trend was seen for women but was not significant for women after adjusting for disability (Fig. 4). Lack of spousal support increased the mortality hazard by 67% in men and 71% in women. Disability significantly increased the mortality hazard in both men and women independent of age. Mortality hazard was not significantly influenced by education and socioeconomic status. There was no significant interaction between the sociodemographic covariates (spousal support, education, socioeconomic status) or disability and SRH in predicting mortality in either men or women.

## SRH as a marker

Can a self-rating of one's own health be a valid marker of health? Given identical true health levels, will two individuals necessarily rate their health with identical response options? When an individual is asked to self-rate his health with a discrete response on an ordinal scale, the response is analyzed with the assumption that it represents his ‘true’ health measured on an underlying latent interval scale. The individual uses some response category cut-points that are unknown to the researcher to categorize his discrete response. For such analysis, the tendency is to assume that all individuals use the same cut-points. However, there is a large body of evidence to suggest that individuals and groups interpret and choose response categories very differently. Two individuals or groups of individuals with identical health levels may rate their own health differently or vice versa, based on their understanding, expectation, and experience of their own health (45). This difference in reporting style referred to as reporting heterogeneity unless recognized and corrected for can lead to misleading comparisons (46). Anchoring vignettes is a strategy used in recent years to identify and overcome the problem of reporting heterogeneity in self-rating responses (47). It has increasingly been used in the last decade to improve interpersonal comparability of self-rating responses in the areas of political efficacy, work disability, job and life satisfaction, and health and health system responsiveness (48–52).

Our study administered vignettes for each of the eight health domains separately as it was felt that the single SRH question is too complex and multidimensional for a concept to be captured by a brief vignette. Our study uses the HOPIT modeling approach to combine information from the anchoring vignettes for mobility and cognition to identify and estimate the response category cut-points and then correct the self-rating response for reporting heterogeneity (17). Our study showed strong evidence of reporting heterogeneity in their self-rating responses largely driven by age, sex, and socioeconomic status. Individuals with higher socioeconomic status and higher education significantly lowered the response category cut-points for cognition, that is, they were more likely to be ‘demanding’ in self-rating their cognitive ability compared to lower socioeconomic status and less educated respondents. After correction for reporting heterogeneity, women, older individuals and those from lower socioeconomic background, were significantly more likely to report greater difficulty in mobility. A similar pattern was seen for cognition self-rating but was not significant.

## Large area–small area comparison of SRH estimates

The national SAGE survey, though rich in information, lacked adequate precision at the district or sub-district level and is of limited value for local micro-planning and resource allocation. On the contrary, the demand for district- or sub-district-level information has greatly increased due to decentralized health micro-planning and decision making in India. Small area estimation are a broad range of statistical techniques that borrows strength by using information about the variable from other similar or related small areas or from information in the same area collected in the past, and thus effectively increase the sample size at the small area level. This information is then combined into the estimation process through a model that links the related small areas through the use of auxiliary information (most often census information) that is available at the small area level (18). Small area estimation has been used to estimate small area disease burden (53–56), disability (57), unmet needs (58), vaccine coverage (59), identify communities at risk for targeted health interventions (60–63) and for understanding geographical disparities, income inequity and poverty (64, 65). The two main challenges of small area estimation are calculating the estimate with any level of precision given the small sample size at the small area level and estimation of its prediction error and there is no consensus on which small area estimation technique provides the most precise estimate with the smallest prediction error. There are few studies that compare small area estimates derived indirectly from large area surveys with those derived directly from small area surveys.

**Fig. 5:**
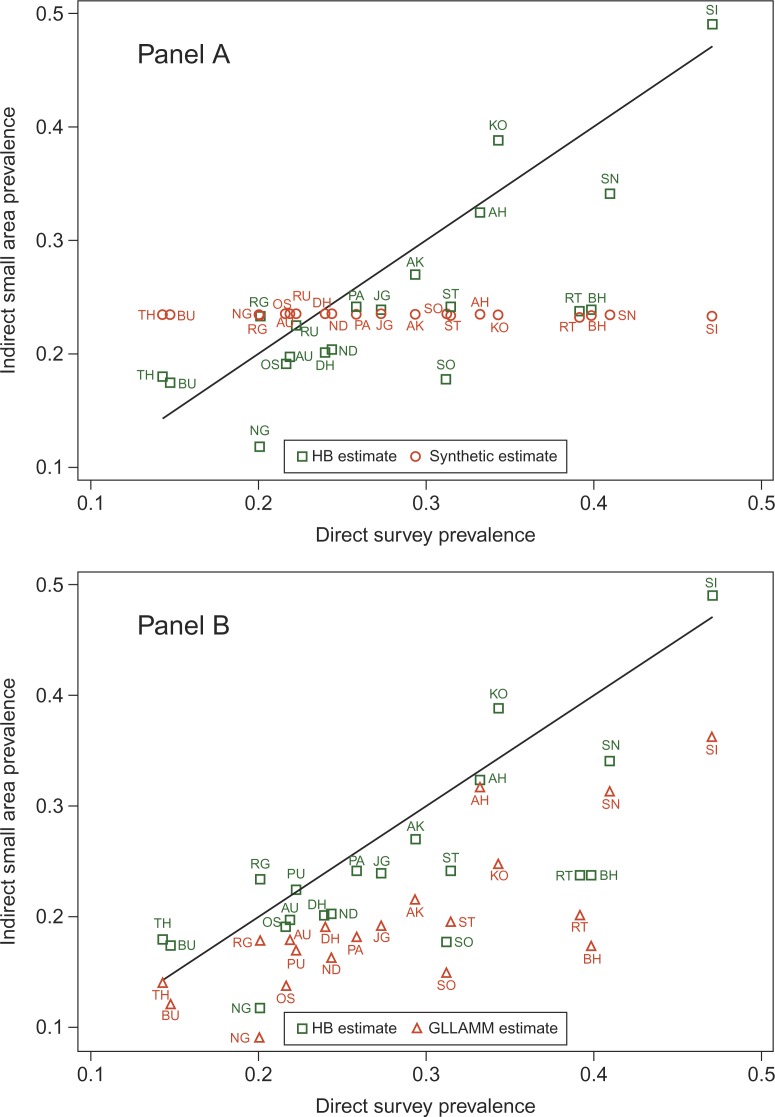
Comparison of HB and indirect synthetic estimate (panel A), and HB and GLLAMM estimate (panel B) of prevalence of good SRH with direct survey estimate for districts in Maharashtra, India. Districts are labeled by their codes. Solid line indicates perfect correlation with direct survey estimate.

We derived ‘good SRH’ prevalence for each district of Maharashtra and for the Vadu community at the sub-district level using four different small area estimation techniques, namely, indirect synthetic estimate based on an age–sex fixed effects model; best linear unbiased linear prediction (BLUP) estimates derived using two routines – xtmelogit and gllamm; and the hierarchical Bayes (HB) estimate, based on a multilevel model. We then compared these small area estimates with the direct weighted survey estimate. The state-level SRH prevalence was 23% (95% credible interval: 20–27%). The district-level SRH prevalence ranged from 5 to 47% with wide intervals reflecting the small sample size at the district level. The district-level indirect synthetic estimate was about 23% with minimal variation between the districts. The district-level BLUP and the HB estimates ranged from 8 to 38%. The HB estimates had wider intervals compared to the BLUP estimates. The correlation between the BLUP and HB estimates was 0.95 while that between the BLUP/HB estimates and the direct survey estimate was 0.75 (Fig. 5). The direct survey estimate of ‘good SRH’ prevalence in the Vadu community was 50%, the BLUP and HB estimates were 46%, whereas the indirect synthetic estimate was 23%. Our study shows that the indirect synthetic estimate though intuitive and easy to derive was imprecise as it assumed that the difference in the SRH prevalence was solely due to age–sex differences in the district populations (66). This assumption is incorrect as SRH is known to vary by contextual factors operating at the area level (67). On the other hand, the multilevel model allowed contextual factors to influence the SRH estimates and provide increased accuracy of standard errors. The BLUP estimate is relatively robust to variations in the sample size of each small area as the model estimates based on fewer observations are ‘shrunk’ towards the global mean for the data (68). BLUPs are a useful smoothing tool. The shrinkage property keeps them from over fitting the data. On the other hand, the HB approach treats both the fixed effects and random effects parameters as random and assumes a joint distribution for these parameters. Modeling is carried out in several stages that are easier to understand even if the model fitting process is complicated. HB estimates have smaller mean square errors and account for the uncertainty in the prediction error than corresponding BLUP estimates. However, the HB estimates are computationally complex and are sensitive to the specification of their priors and use Markov Chain Monte Carlo simulations to approximate the posterior distributions of the parameter estimates.

## Critiques and debates

Despite its widespread use as a measure of health and its established value as a predictor for adverse health outcomes, SRH as a marker of the individual's true health often tends to be viewed by many with skepticism. This skepticism is underpinned in the larger debate between perceptions versus observation; between the emic and the etic perspective. The prevailing view, largely influenced by a commentary by Amartya Sen in the British Medical Journal in 2002, is that self-reports of illness and health are misleading, as socially disadvantaged individuals from low- and middle-income settings fail to perceive and report illness or health deficits because an individual's assessment of their health is directly contingent on their social experience (69). Sen, therefore, argues that perceptions and self-reports of health (the emic perspective) can be misleading and obfuscate the true extent of health deficit that is more likely to be captured through external more objective assessments (etic perspective). The authors of another study that could not detect a social gradient in a mother's report of her child's diarrhea also argued that reported measures of morbidities are misleading (70). The counterview is that SRH as a marker of true health is valid and of value by itself, as it is the perception that largely shape and determine an individual's health behavior and health seeking behavior. However, the concern with SRH is not its validity in itself but its interpersonal comparability (12). It is therefore essential to identify and correct for the reporting heterogeneity inherent in any self-rating response before making any comparisons.

Another area of debate has been the reference time for the self-assessment questions. A global measure of health should ideally reflect a cumulative measure of the individual's health and hence by implication should be insensitive to short-term changes in health. If the time reference for the global health question is short as in our study wherein we asked ‘In general, how would you rate your health, today?’, the response is likely to be affected by short-term fluctuations in health like mild illness or even some cyclical variation in wellbeing like menstruation. This can add noise in the ability of SRH to predict long-term outcomes such as mortality. Researchers have tried to address this concern by specifying different time anchors, say a month, for the SRH question while others have used different terms – perceived general health which is more global in its meaning and predicts long-term health outcomes to distinguish from perceived current health that predicts health care utilization and medication use (71). A stable alternative may be to ask the SRH questions with a broader time frame – maybe a week or a month, though this is a topic for further research.

## In a nutshell … so, what next?

This thesis establishes the value and utility of including SRH questions as a measure of health and predictor of mortality within survey settings or demographic surveillance systems in the context of aging in India. It advocates the use of such a simple measure in survey settings to identify vulnerable communities for targeted health interventions, plan and prioritize resource allocation, and evaluate health interventions in resource-scarce settings. It provides evidence to promote social policy and program interventions aimed to increase social networking and social participation especially among those socially disadvantaged and elderly. The thesis highlights the need to identify and correct SRH responses for reporting heterogeneity to improve its interpersonal comparability. Finally, it demonstrates the potential of using information from large national surveys for planning and evaluation of policies and programs at the district and sub-district level.

This thesis also raises and leaves unanswered questions – especially questions on the sensitivity of SRH to gradual changes in health state. How does the individual's SRH response change with gradual deterioration of his health over a long period? How quickly or slowly does it change? How much does health need to decline or detrimental factors need to cumulate to effect a change in the SRH response? What determines or triggers this change? Is a change in SRH a better predictor for adverse health outcomes? How does an individual's expectation of his health modulate the effect of his health experiences on SRH? How do experiences and expectations interplay to generate the considered SRH response? How and why do contextual factors influence SRH? How can the interpersonal and cross-cultural comparability of SRH be further improved? These questions are crucial to the understanding of SRH and some may be answered when the second round of SAGE is implemented in the same cohort.

Finally, I hope this thesis provides the basis and impetus to generate and integrate similar and harmonized adult health and aging data platforms within demographic surveillance systems in different regions of India and elsewhere. I hope it raises awareness and stimulates the scientific community and policy makers to prioritize and mainstream successful aging into the national research and development agenda of low- and middle-income countries that are witness to rapidly aging populations.

## Appendix

Summary of measures included in the full version of the SAGE survey (adapted from source: http://www.who.int/healthinfo/sage/SAGE_Waves0_1_SummaryMeasures.pdf)

**Table:**

Domain	Household measures
Household identification, contact and sampling details	Identification and contact details; structure of household; dwelling characteristics; improved water, sanitation and cooking facilities
Transfers and support Networks	Family, community, and government assistance into and out of the household; informal personal care provision/receipt
Assets, income and Expenditure	List of household assets; sources and amount of household income; improved household expenditure on food, goods and services, health care
Household care and health insurance	Persons in household needing care; mandatory and voluntary health insurance coverage
**Domain**	**Individual measures**
Sociodemographic characteristics	Sex, age, marital status, education, ethnicity/background, religion, language spoken, area of residence, employment, and education of parents
Work history and benefits	Length of time worked, reasons for not working, type of employment, mode of payment, hours worked
Health states and descriptions	Overall self-rated health; eight self-rated health domains (affect, mobility, sleep/energy, cognition, interpersonal activities, vision, self-care and pain); 12-item WHO Disability Assessment Schedule, Version 2 (WHODAS-II); activities of daily living (ADLs); instrumental activities of daily living (IADLs); vignettes on health state descriptions
Anthropometrics, performance tests and biomarkers	Measured blood pressure; self-report and measured height and weight; measured waist and hip circumference; timed walk; near and distant vision tests; grip strength, executive functioning (verbal recall, digit span forwards and backwards, verbal fluency); spirometry; non-fasting finger prick blood sample (stored at -20C) as dried blood spots
Risk factors and preventive health behaviors	Smoking, alcohol consumption, fruit and vegetable intake, physical activity (GPAQ)
Chronic conditions and health services coverage	Self-reported and symptomatic reporting of arthritis, stroke, angina (Rose Questionnaire), asthma, and depression (ICD-10, DSM-IV). Self-reporting of diabetes, chronic lung disease, hypertension, cataracts, oral health, injuries, and cervical, and breast cancer screening
Health care utilization	Past need for health care, reasons for health care or for not receiving health care, inpatient and outpatient health care: number of admissions/visits within the past 3 years (inpatient) or 1 year (outpatient), reasons for admission/visit, details of hospital or provider, costs of hospitalization or health care visit, satisfaction with treatment, health system responsiveness, vignettes for responsiveness of health services
Social cohesion	Community involvement and social networks, perceptions of other people and institutions, safety in local area, stress, interest in politics and perceptions of government
Subjective wellbeing and quality of life	Perceptions about quality of life and wellbeing, 8-item WHO Quality of Life measure (WHO-QOL), Day Reconstruction Method (DRM)
Impact of caregiving	Household members needing care, type of care required, length of time spent on care, costs of care, impact of providing care on career wellbeing
